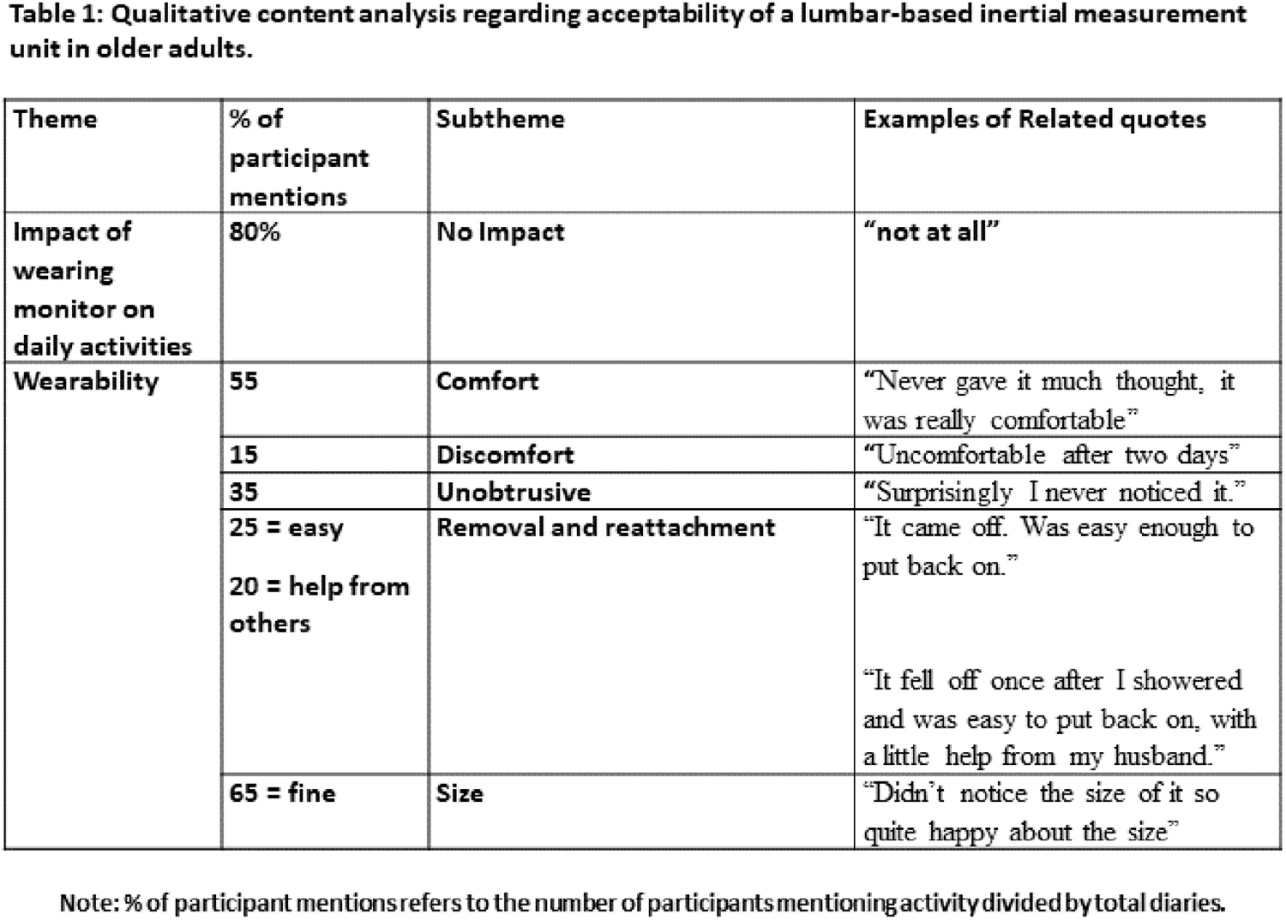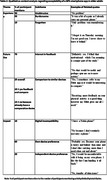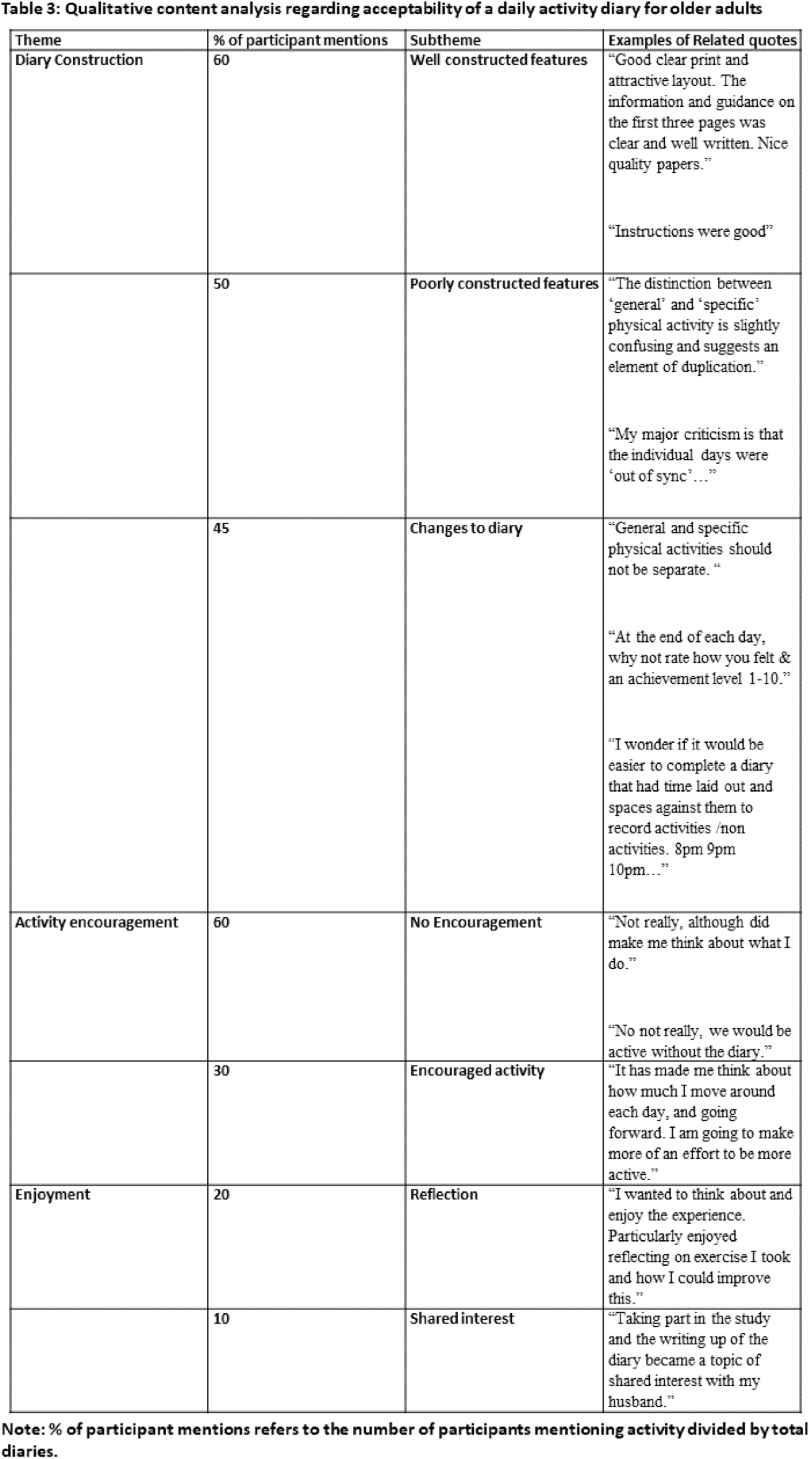# Feasibility and acceptability of a continuous remote activity monitoring protocol in older adult dyads: A mixed methods pilot study

**DOI:** 10.1002/alz.094327

**Published:** 2025-01-09

**Authors:** Ríona Mc Ardle, Jenny L Wales, Calum Alexander Hamilton, Leigh James Ryan, Louis McCarthy, Silvia Del Din, Sayeh Bayat, Gro Gujord Tangen, Neil Ireson, Vita Lanfranchi, Nicolas Farina, Ben Hicks

**Affiliations:** ^1^ Newcastle University, Newcastle Upon Tyne UK; ^2^ Newcastle University, Newcastle‐Upon‐Tyne, Tyne and Wear UK; ^3^ Newcastle University, Newcastle upon Tyne UK; ^4^ Hotchkiss Brain Institute, Calgary, AB Canada; ^5^ Norwegian National Centre of Ageing and Health, Sem Norway; ^6^ University of Sheffield, Sheffield UK; ^7^ University of Plymouth, Plymouth UK; ^8^ Brighton and Sussex Medical School, Brighton UK

## Abstract

**Background:**

Walking is a key facilitator of healthy ageing and may reduce risk of cognitive decline in older adults. To develop suitable, accessible interventions, we must objectively consider the socio‐ecological factors which influence participation in walking activities. For example, walking may be influenced by the volume and type of activities one’s partner participates in (i.e., dyadic interactions), or the walkability of their local area. Wearable technologies can continuously and remotely capture digital walking outcomes, such as volume, pattern, variability and location of activities. This pilot study aimed to explore the feasibility and acceptability of deploying a continuous remote activity monitoring toolkit in older adult dyads (i.e., couples).

**Methods:**

Participants were asked to engage with three forms of remote activity monitoring over a period of seven days: (1). Wearing an inertial measurement unit (IMU; AX6, Axivity) on their lower backs, (2). Carrying a smartphone installed with a GPS app on excursions outside the home, and (3). Completing an activity diary (e.g., daily journeys, motivations/perceptions of journeys) each night. Upon study completion, participants were asked to complete open‐ended questionnaires regarding their experiences of the protocol. Feasibility was assessed by quantitatively calculating completion of each form of activity monitoring, while qualitative content analysis of the questionnaires was employed to understand the acceptability of the protocol.

**Results:**

21 dyads (n = 42) participated in the study (Age (median (range)): 69 (61‐79)). 95% of participants wore the IMU for seven days (5% removed early for holidays). 100% completed their activity diaries. 77% (n = 226) of all data collection days (n = 294) were captured from the GPS app; reasons for data loss (68 days) include possible technical error (69.12%), not leaving the house (29.41%), or forgetting the GPS device (1.47%). Most participants found all forms of activity monitoring acceptable; common themes are reported in Tables 1‐3.

**Conclusion:**

Results suggest that a protocol of continuous remote activity monitoring using digital devices and an activity diary is feasible and acceptable to older dyads. Further work will explore how data acquired can be used to identify socio‐ecological predictors of walking and examine independence/interdependence in walking between members of each dyad.